# The Resolution of a Biopsy-Proven Enchondroma in the Proximal Humerus Over a 14-Year Interval

**DOI:** 10.7759/cureus.93194

**Published:** 2025-09-25

**Authors:** Gilbert Lanoue, Eva Wasyliw, Jennifer Adams, Felix Gonzalez, Christopher Wasyliw

**Affiliations:** 1 Department of Diagnostic Radiology, AdventHealth Hospital, Orlando, USA; 2 High School, Trinity Preparatory School, Winter Park, USA

**Keywords:** bone scan, chondrosarcoma, enchondroma, mri, regression, resolution

## Abstract

Enchondromas are commonly encountered benign cartilaginous tumors that, in most cases, remain stable when followed over time. The loss of cartilaginous matrix is unusual, requiring further investigation into potential regression or malignant degeneration. A few case reports in the literature describe enchondroma regression, but to our knowledge, in the literature in English, only one other case report has shown complete resolution on X-ray. We present an unusual case of complete resolution of a biopsy-proven enchondroma on MRI and bone scan, which contributes to the sparse literature on the natural regression of enchondromas.

## Introduction

Enchondromas are benign intramedullary neoplasms composed of hyaline cartilage, most commonly arising in the metaphyseal regions of long bones such as the femur, humerus, and tibia [1]. They account for 3-17% of all bone tumors and approximately 20% of all cartilage tumors [1]. Although enchondromas can occur at any age, they are most frequently diagnosed between the second and fourth decades of life. Radiographically, they often demonstrate a chondroid matrix with characteristic punctate or ring-and-arc calcifications. On bone scintigraphy, they typically demonstrate mild activity, while MRI shows classic features including T2 hyperintensity, T1 hypointensity, and post-contrast enhancement with low-signal internal foci. Most lesions remain stable over time, with some showing minimal growth or, in rare cases, regression [1].

In clinical practice, enchondromas are often discovered incidentally on imaging performed for unrelated conditions. However, distinguishing them from low-grade chondrosarcomas (atypical cartilaginous tumors) can be challenging, both radiographically and histologically [2]. Chondrosarcomas comprise 20-30% of all bone sarcomas, with peak incidence between 40 and 60 years of age [3]. While solitary enchondromas have a relatively low risk of malignant transformation (up to 4.2%) [4], patients with multiple enchondromatosis (Ollier’s disease) face a significantly higher lifetime risk, estimated at up to 25% [5]. Worrisome clinical and imaging features suggestive of malignant degeneration include persistent or progressive pain, endosteal scalloping or erosion, rapid interval growth, loss of mineralization, and the presence of soft-tissue extension [6].

Regression of enchondromas with loss of mineralization replaced by normal medullary fat is unusual and poorly understood, with complete resolution even more rare [7,8]. When loss of mineralization is observed on follow-up imaging, further evaluation with cross-sectional imaging or biopsy is often warranted to differentiate between true regression and early malignant transformation. The current case adds to the limited body of evidence on enchondroma regression and underscores the need for careful surveillance and ongoing research to better understand the biological behavior of these lesions.

## Case presentation

**Figure 1:**
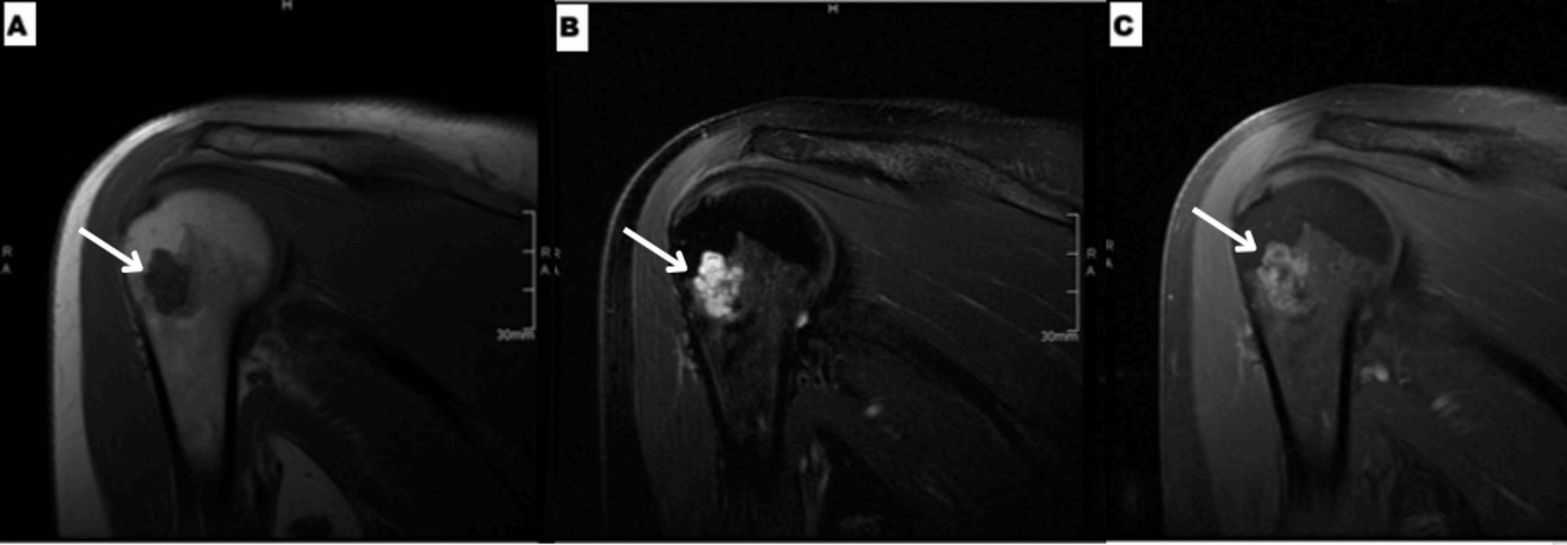
MRI findings - 1 2009 coronal T1 (A), coronal T2 fat sat (B), and coronal T1fat sat post contrast (C) MRI images show a well circumscribed, non-aggressive mildly lobulated lesion in the humeral head and neck central medullary space which is low in signal on T1, predominantly bright on T2 fat sat with internal low signal foci and shows mild predominant peripheral enhancement without endosteal erosion or perilesional marrow edema (arrows) MRI: magnetic resonance imaging

**Figure 2:**
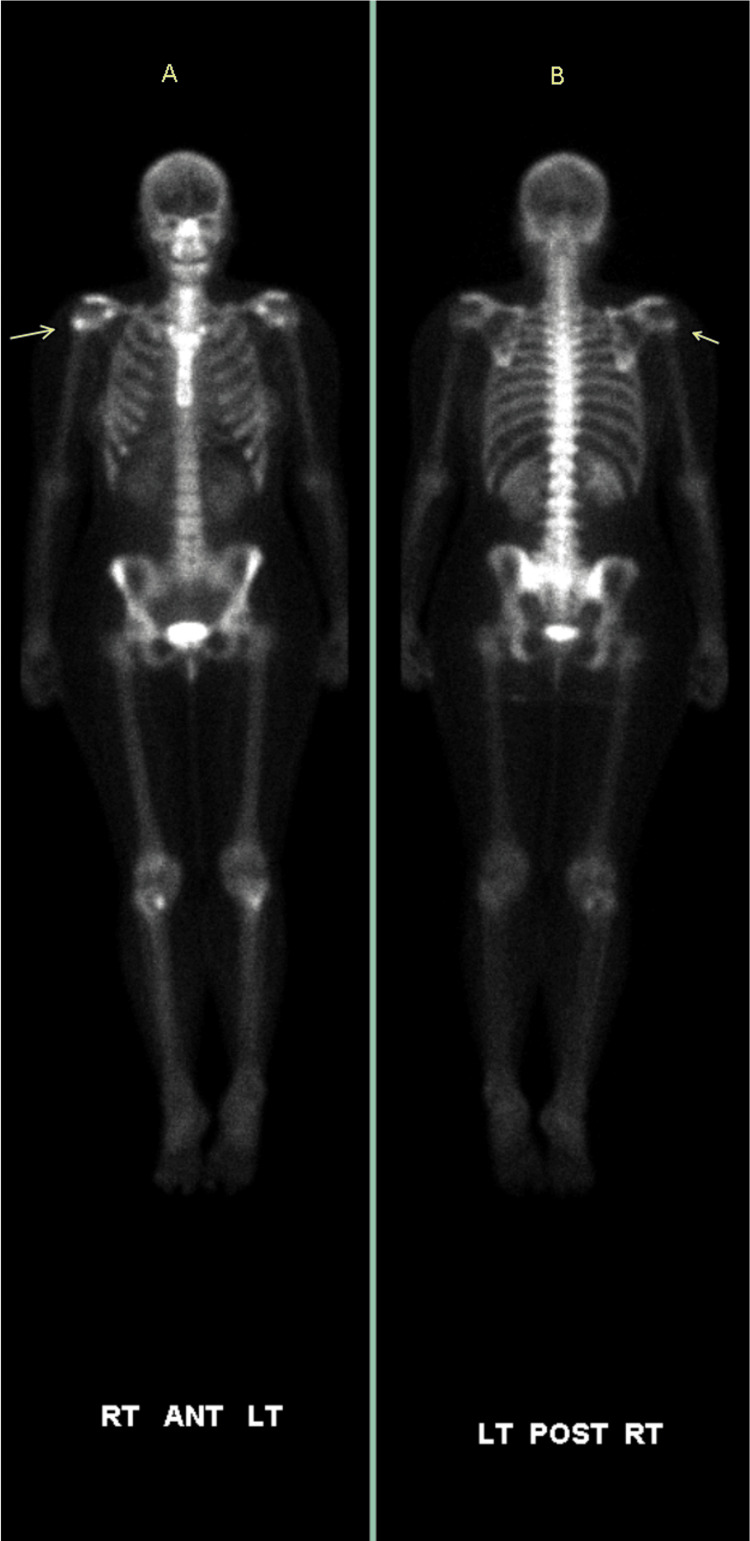
Bone scan findings - 1 Tc 99m nuclear medicine anterior (A) and posterior (B) whole body bone scan shows nonspecific activity in the right humeral neck in 2009 (arrows)

A 46-year-old female with a history of melanoma and intracranial meningioma presented to her orthopedic surgeon in 2009 with vague pain in her right shoulder. She had no history of injury, overuse, or systemic musculoskeletal symptoms. Given her oncologic history and persistent shoulder discomfort, she was referred to our institution, and an MRI and bone scan were performed to further evaluate the etiology of her symptoms. MRI of the right shoulder showed a small cartilaginous lesion in the proximal humeral metaphysis. The lesion measured 1.7cm with low signal on T1- and bright on T2-weighted sequences with low signal foci within it (Figure 1). There was no cortical involvement, perilesional marrow edema, or associated soft tissue mass. Post-contrast imaging showed thin peripheral enhancement. X-rays were not available or obtainable from the referring provider outside our facility. A concurrent bone scan shows mild radiotracer uptake in the same region, correlating with the MRI findings (Figure 2). The imaging appearance was described as a low-grade cartilaginous lesion.

Due to the patient’s symptoms of pain, history of melanoma, and clinical desire to exclude a low-grade chondrosarcoma, she underwent a biopsy of the lesion on October 3, 2009. Histopathology confirmed a benign enchondroma, composed of hyaline cartilage interspersed with medullary hematopoietic elements and trabecular bone (not available due to chronicity). Given the benignity of the lesion, interval imaging surveillance was recommended. However, she was lost to follow-up for over 14 years.

**Figure 3:**
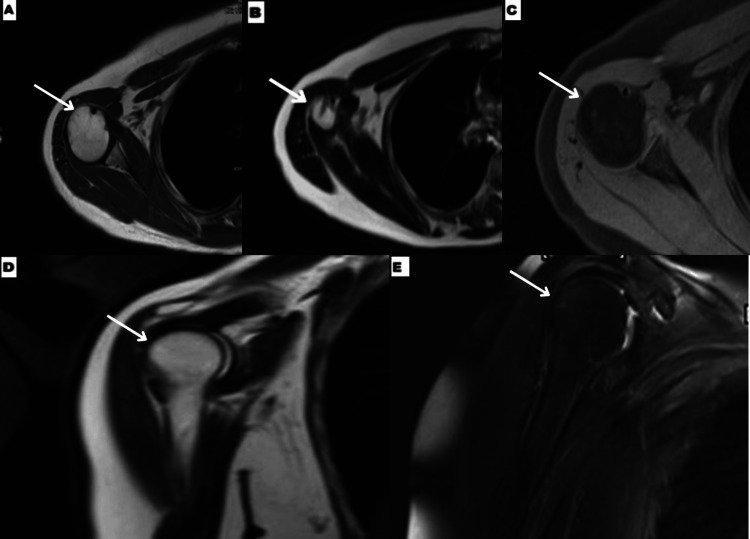
MRI findings - 2 MRI of the humerus on 2/29/24 with axial T1 (A, B), axial T1 fat sat post contrast (C), cor T1 (D), and cor T2 fat sat (E) images show complete resolution of prior intramedullary lesion (A, C, D, E arrows). Axial T1 (B) shows residual biopsy tract in the humeral neck (arrow) MRI: magnetic resonance imaging

**Figure 4:**
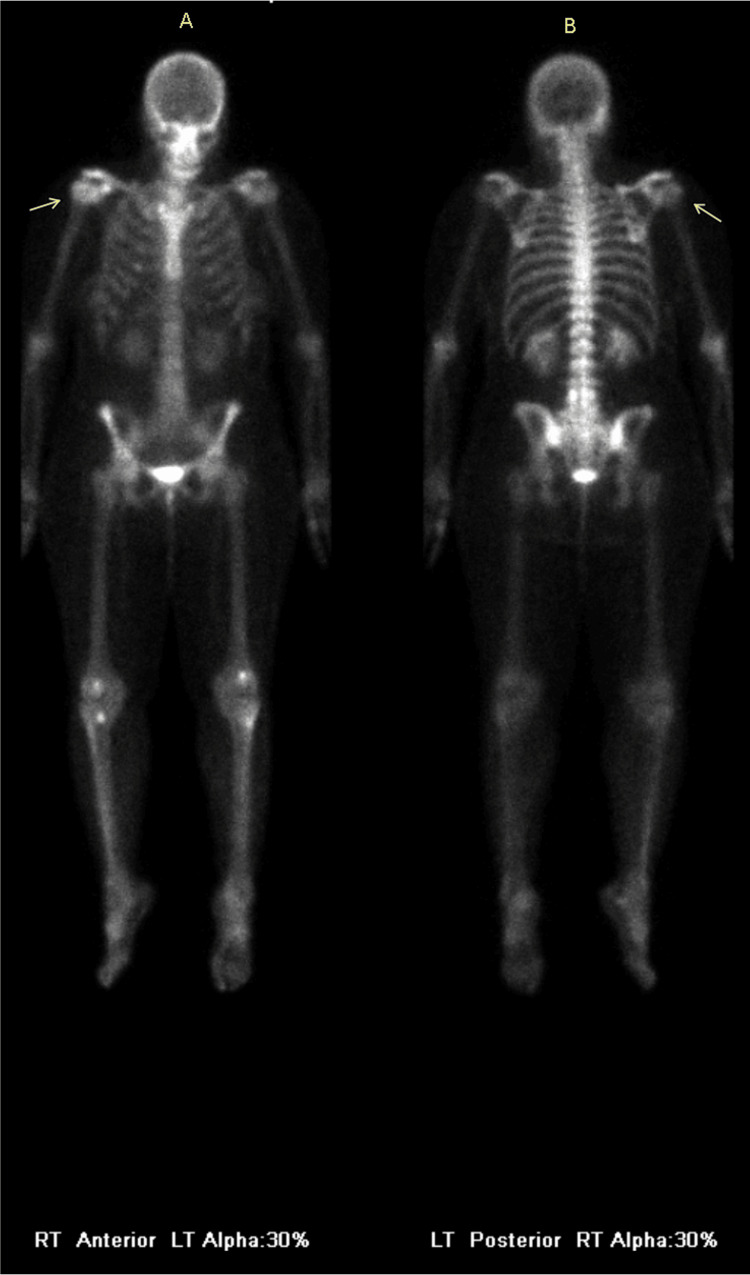
Bone scan findings - 2 Tc 99m nuclear medicine anterior (A) and posterior (B) whole body bone scan shows complete resolution of activity in the humeral neck in 2024 (arrows)

In 2024, the patient re-established care for follow-up imaging. She reported no ongoing pain or functional limitation in her right arm or shoulder. Follow-up MRI and bone scan both showed complete resolution of the lesion (Figures 3, 4). On MRI, the axial T1 series reveals a thin linear tract near the humeral neck (Figure 3b), consistent with the prior biopsy site, but the bone marrow signal was otherwise normal. There was no mass, cortical abnormality, or any sign of residual disease. The patient remained asymptomatic and functional throughout this 14-year interval. 

## Discussion

Although enchondromas are generally regarded as indolent and stable lesions, our case demonstrates that regression and even complete resolution are possible, challenging the traditional understanding of their natural history. The notion that enchondromas may regress is supported by limited prior reports in the literature. One of the earliest examples was described by Baruchin et al. in 1981 [8], who reported a pathologic fracture through an enchondroma in the fifth finger that resolved with conservative splinting. Follow-up radiographs revealed restoration of the medullary cavity to its normal appearance, though MRI was not available at that time to confirm fat replacement or matrix resorption. In that case, as in ours, mechanical or traumatic insult to the cartilaginous matrix appeared to act as a catalyst for subsequent lesion resolution.

The mechanisms underlying the regression or resolution of enchondromas remain poorly understood, though several hypotheses have been proposed. One potential pathway involves post-biopsy or post-trauma necrosis of the cartilaginous matrix, followed by gradual replacement with marrow fat. Internal ischemic events may similarly result in chondrocyte death, after which osteoclasts resorb the necrotic cartilage and osteoblasts facilitate trabecular reconstitution, ultimately restoring a normal marrow cavity [9]. This sequence of cellular turnover mirrors physiologic remodeling, suggesting that enchondroma regression may represent an exaggerated or pathologically triggered form of normal bone-healing processes.

Additional insights come from Sensarma et al., who described regression but not full resolution of a proximal humeral enchondroma in a 50-year-old woman with a history of thyroid cancer treated by thyroidectomy and radioactive iodine [10]. They proposed a mechanism akin to endochondral ossification, whereby chondrocytes differentiate into hypertrophic chondrocytes that mineralize the extracellular matrix, creating an environment conducive to vascular invasion. This vascularization recruits chondroclasts and osteoclast progenitors that degrade cartilage, while osteoblast precursors deposit non-mineralized bone matrix. Mature osteoblasts subsequently mineralize this matrix with calcium hydroxyapatite, and osteoclasts participate in shaping the developing trabecular bone and marrow cavity. Through this cascade, cartilage is gradually resorbed and replaced with normal marrow fat. While the precise trigger remains uncertain, the prior history of radioactive iodine exposure in their patient raises the possibility that systemic or treatment-related factors may accelerate or facilitate enchondroma regression.

Our case is distinct in that it documents the complete resolution of an enchondroma, supported by multimodality imaging, including MRI and bone scintigraphy, as well as tissue sampling across a prolonged 14-year follow-up interval. To our knowledge, such comprehensive longitudinal documentation is exceedingly rare in the literature. This prolonged observation period allows greater confidence that the observed regression represents a true biological process rather than an imaging artifact, sampling error, or incomplete lesion characterization.

The clinical significance of regression lies in its diagnostic implications. While enchondromas are typically stable, the loss of cartilaginous matrix on serial imaging is traditionally viewed with suspicion, as it can suggest malignant degeneration into chondrosarcoma. Features such as progressive pain, rapid growth, endosteal erosion, and soft-tissue extension further raise this concern [6]. However, as demonstrated in our case, loss of mineralization may also result from benign processes such as fatty replacement or endochondral ossification, and therefore does not always indicate malignant transformation. This underscores the importance of correlating imaging findings with clinical context, tissue sampling, and longitudinal follow-up when evaluating suspected regression.

Ultimately, our report contributes to the growing body of evidence that enchondromas are biologically more dynamic than previously appreciated. While the majority remain stable, a subset may regress or even resolve completely, through mechanisms involving necrosis, ischemia, or ossification pathways. Recognition of this phenomenon is important for radiologists, pathologists, and orthopedic oncologists alike, as it broadens the differential diagnosis for changing enchondroma morphology and highlights the need for nuanced interpretation when mineralization loss is encountered.

Due to sparse knowledge on the biological behavior of these tumors, guidelines for active surveillance are lacking, and follow-up strategies vary amongst institutions [6]. The management of enchondromas and low-grade chondrosarcomas (atypical cartilaginous tumors) highlights the significance of radiographic monitoring, particularly for asymptomatic lesions. Serial follow-up is recommended over immediate curettage for non-painful cartilaginous lesions, regardless of size, due to the challenges associated with imaging criteria for both entities. Small (<5 cm) asymptomatic intraosseous cartilaginous lesions should be observed as benign enchondromas without the need for surgical intervention. This conservative approach is supported by findings indicating that only a small percentage of cases require surgical removal due to invalidating pain or radiographic changes, emphasizing that many lesions may not necessitate aggressive treatment [11].

For effective follow-up, asymptomatic enchondromas larger than 5 cm benefit from annual MRI examinations, while smaller lesions should undergo annual clinical evaluations and bi- or triennial radiological assessments. The time between the initial diagnosis of enchondroma and any malignant transformation can vary significantly, suggesting that lifelong radiological follow-up may be warranted. Given these considerations, conservative management should be prioritized for asymptomatic enchondromas, with annual MRI monitoring, particularly for lesions exceeding 5 cm. If no changes are observed after two years, the frequency of imaging can be adjusted to every two to three years, underscoring the need for a standardized follow-up protocol to ensure accurate monitoring and timely intervention when necessary [11].

To ensure accurate diagnosis, clinicians should prioritize correlating regression findings with the patient's clinical context, including their medical history and any symptoms. This comprehensive approach, integrating imaging results, clinical evaluations, and, when necessary, tissue analysis, will help distinguish benign changes from potential malignancy, thereby reducing the risk of unnecessary interventions and alleviating patient anxiety. Continued reporting of such cases will be essential to further elucidate the biological mechanisms and clinical implications of enchondroma regression.

## Conclusions

The spontaneous resolution of enchondromas, while rare, is possible. The probable mechanisms for involution include necrosis and fatty replacement or endochondral ossification.
